# Detection of Filarial IgG and IgM Antibodies Among Individuals With Lymphedema in the Kamwenge District, Western Uganda

**DOI:** 10.7759/cureus.87532

**Published:** 2025-07-08

**Authors:** Vicent Mwesigye, Joanita Berytah Tebulwa, Benson Musinguzi, Bosco Bekita Agaba, Charlse Nkubi Bagenda, Francis Bajunirwe, Joel Bazira, Edgar Mulogo, Itabangi Herbert, Frederick Byarugaba

**Affiliations:** 1 Department of Medical Laboratory Sciences, Faculty of Medicine, Mbarara University of Science and Technology, Mbarara, UGA; 2 Department of Nursing, Faculty of Medicine, Mbarara University of Science and Technology, Mbarara, UGA; 3 Department of Medical Laboratory Sciences, Faculty of Health Sciences, Muni University, Arua, UGA; 4 Department of Community Health, Faculty of Medicine, Mbarara University of Science and Technology, Mbarara, UGA; 5 Department of Microbiology and Parasitology, Faculty of Medicine, Mbarara University of Science and Technology, Mbarara, UGA; 6 Department of Microbiology and Immunology, Faculty of Health Sciences, Busitema University, Mbale, UGA; 7 Department of Microbiology, Faculty of Medicine, Mbarara University of Science and Technology, Mbarara, UGA

**Keywords:** filarial igg and igm antibodies, kamwenge uganda, lymphatic filariasis, lymphedema, podoconiosis

## Abstract

Filarial infections trigger a complex immune response characterized by the production of different antibodies, particularly immunoglobulin G (IgG) and immunoglobulin M (IgM). These immunoglobulins play a key role in diagnosing the disease, with IgM typically indicating recent infection and IgG reflecting past or ongoing exposure. Assessing their presence provides valuable insight into an individual's immune response and infection history. This study examined the levels of IgG and IgM in people living with lymphedema in the Kamwenge district, Western Uganda, to better understand their immunological status in relation to filarial infection.

This cross-sectional study, conducted in the Kamwenge district, aimed to assess the presence of anti-filarial antibodies among lymphedema patients. A total of 154 participants, predominantly female (71.4%), with a mean age of 54.7 years, were selected through simple random sampling. Serological testing using the Abbexa Filariasis IgG/IgM Rapid Test revealed that 10.4% tested positive for IgG, and 1.9% for IgM antibodies. We enrolled a total of 154 participants, the majority of whom were female 110 (71.4%) while 44 (28.6%) were male. The participants had a mean age of 54.7 years, with a standard deviation of 15.6 years. Overall, 10.4% (n=16) tested positive for filarial antibodies. Specifically, 10.4% (n=16) were positive for filarial IgG, while 1.9% (n=3) tested positive for IgM antibodies. The serological findings demonstrated a low prevalence of recent filarial infections, with a higher occurrence of past or chronic exposure among participants. This suggests that while active transmission may be limited, lymphatic filariasis remains an ongoing public health concern in the Kamwenge district. These results emphasize the need for continued surveillance, early detection, and targeted interventions to effectively manage and mitigate the burden of filarial-related lymphedema in the region.

## Introduction

Lower limb lymphedema, commonly known as "elephantiasis," is a chronic condition caused by dysfunction of the lymphatic system, resulting in the accumulation of localized fluid [1,2]. It is characterized by the accumulation of lymphatic fluid in the interstitial tissues due to a malfunctioning lymphatic system [3,4]. Despite its debilitating nature, lymphedema remains insufficiently addressed in scientific research, medical education, and clinical practice. The build-up of protein-rich fluid in the interstitial tissues due to inadequate lymphatic drainage can lead to complications such as fibrosis, fat deposition, and various skin abnormalities, including papillomas, dermal thickening, and acanthosis [4]. Lymphedema is generally categorized as either primary or secondary, based on its origin. While secondary lymphedema results from external damage or disease affecting the lymphatic system, primary lymphedema arises from inherent structural or functional lymphatic abnormalities [5]. Around 70% of primary lymphedema cases are idiopathic with no currently known genetic cause [5]. However, the remaining 30% are linked to genetic mutations, with approximately 20 genes implicated in lymphatic dysfunction [6]. This condition is also a feature of certain genetic syndromes, including Turner syndrome, Noonan syndrome, tuberous sclerosis, and capillary malformation-arteriovenous malformation syndrome [7]. Currently, there is no definitive cure for primary or secondary lymphedema. Treatment primarily aims to manage symptoms and prevent complications [7,8]. Importantly, the approach to prevention and treatment depends on identifying the specific underlying cause.

The immune response to filarial infection is complex and involves the production of various immunoglobulins, notably immunoglobulin G (IgG) and immunoglobulin M (IgM) [9,10]. The detection of these antibodies serves as a crucial diagnostic tool for identifying active or past infections and understanding the immune status of individuals affected by filariasis [11]. IgM antibodies typically indicate a recent infection, while IgG antibodies suggest either chronic infection or past exposure to the filarial antigens. The presence of these antibodies can also provide insights into the underlying pathophysiological mechanisms contributing to lymphedema [12,13].

The diagnosis of lymphedema caused by filarial typically involves clinical assessment and laboratory tests. Traditionally, the diagnosis has relied on the identification of the adult worms or their microfilariae in blood samples [11,14]. Most filarial species exhibit nocturnal periodicity, meaning their microfilariae (larval stage) are primarily found in the peripheral blood during specific hours, often at night (10 PM to 2 AM) [15,16]. This necessitates inconvenient blood collection times, which can be challenging for both patients and healthcare workers in the Kamwenge district. Serological rapid tests to detect IgG and IgM antibodies against filarial antigens are reliable diagnostic alternatives; however, they have not yet been conducted among patients in the Kamwenge district. These serological tests can provide valuable information about the immune status of individuals and facilitate the identification of those at risk of developing lymphedema. Therefore, this study aimed to detect filarial IgG and IgM antibodies among individuals with lymphedema in the Kamwenge district, Western Uganda.

## Materials and methods

Study design, population, and setting

This was a cross-sectional sero-survey. Participants with lymphedema were selected through simple random sampling and included individuals who had lived in the Kamwenge district for more than three months.

Inclusion and exclusion criteria

Participants were included in the study if they exhibited signs of lymphedema, regardless of age, and were either receiving care at Rukunyu Hospital or had been invited from the Kamwenge district community. Eligibility required informed consent from adult participants or emancipated minors, while for children above seven years, both caregiver consent and the child’s assent were mandatory. The study excluded individuals who showed no signs or symptoms of lymphedema, those currently undergoing treatment, and those who did not provide informed consent.

Sample size calculation

The sample size for this study was determined using the Kish and Leslie formula (1965), which is suitable for estimating proportions in cross-sectional studies.

$n = \frac{Z^2_{\alpha/2} \cdot P \cdot (1 - P)}{r^2}$

Here, Z is the standard normal deviate corresponding to a 95% confidence level (Z=1.96); P is the estimated prevalence of lymphedema in the Kamwenge district, which was 34.7% (P=0.347), based on a recent study [4]; and r is the desired margin of error, set at 8% (r=0.08). Substituting these values into the Kish and Leslie formula:

$n = \frac{(1.96)^2 \times 0.347 \times (1 - 0.347)}{(0.08)^2}$

n = 136 participants

To account for potential non-response or incomplete data, we included an additional 10%, resulting in a final minimum sample size of 150 lymphedema patients to be recruited from Rukunyu Hospital.

Laboratory procedures

Sample Collection and Processing

A vein was selected and punctured using a sterile vacutainer needle and holder, and blood was collected into an ethylenediaminetetraacetic acid (EDTA) vacutainer tube. Following standard venepuncture procedures, approximately 2 mL of venous blood was drawn from each participant into a clean, labelled collection tube. The median cubital vein was identified, and the surrounding skin was disinfected by wiping with cotton wool soaked in 70% alcohol and allowing it to air dry. The collected blood samples were then transported to the laboratory, where an immunochromatographic test (ICT) was performed to detect the presence of Filarial IgG and IgM antibodies.

Circulating Filarial IgG and IgM Testing

Two millilitres of blood were collected into EDTA vacutainer tubes, transported to the laboratory, centrifuged, aliquoted, and analyzed for filarial IgG and IgM antibodies using the immunochromatographic test (ICT) with the Abbexa Filariasis IgG/IgM rapid serological test. This assay offers excellent diagnostic accuracy, with a sensitivity of 92.3% for IgG and 98.5% for IgM, and a specificity of 100% for both IgG and IgM, indicating a high level of reliability in detecting both positive and negative cases.

Data management and statistical analysis

Data were double-entered by the principal investigator into Microsoft Excel (Redmond, WA: Microsoft Corp.), which was exported to Stata software version 17 (College Station, TX: Stata Corp LLC) for cleaning and statistical analysis. Age was tested for normality using the Shapiro-Wilk normality test and was normally distributed (p=0.08797). Age was summarized using mean±standard deviation. The mean age in participants with filarial IgG positivity was compared with the mean age in participants without filarial IgG positivity using the Student's t-test. Categorical variables were summarized using frequencies and proportions and compared using Fisher's exact test. A p-value of less than 0.05 was considered statistically significant.

## Results

Out of 154 participants, the majority, 110 (71.4%), were female, while 44 (28.6%) were male. The mean age of the participants was 54.7 (±15.6) years, one (0.6%) were below 10 years of age, six (3.9%) were between 11 and 24 years of age, 45 (29.2%) were between 25 and 49 years of age, and 102 (66.2%) were above 50 years of age. Overall, 10.4% (n=16) tested positive for filarial antibodies. Specifically, 10.4% (n=16) were positive for filarial IgG, while 1.9% (n=3) tested positive for IgM antibodies.

Seventy-nine (51.3%) participants had not attained the primary level of education, 68 (44.2%) had attained the primary level, while eight (7.9%) participants had attained the secondary level of education. Forty-four (29.1%) of the participants were single, while 71 (47.0%) were married, 26 (17.2%) separated or divorced, three (2.0%) cohabiting, and seven (4.6%) widowed.

**Figure 1:**
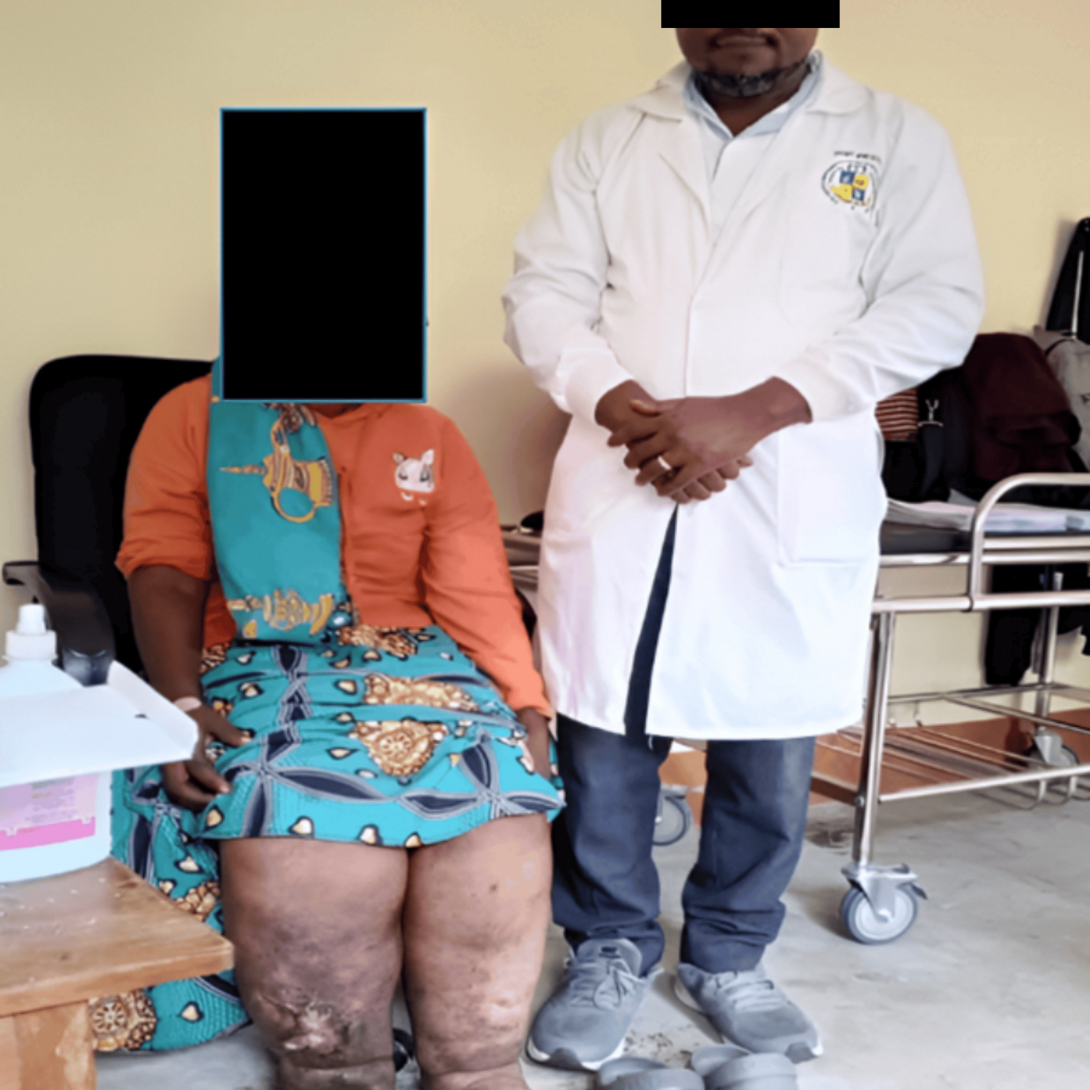
The principal investigator with one of the participants who had lymphedema, an ulcerative wound on her right leg, and cellulitis on both legs.

A total of 132 (85.7%) participants were Bakiga; three (1.9%) were Banyankole; and 19 (12.3%) belonged to other tribes. A total of 114 (75.0%) participants were peasants or farmers engaged in agriculture and grazing, while 137 (91.3%) resided in rural settings. A total of 149 (96.8%) participants had all lower limbs affected, and 16 (11.3%) had discharging wounds (Table 1 and Figure 1).

**Table 1:** Sociodemographic and behavioral characteristics of study participants. *For some variables (marital status, religion, occupation, residence type, and discharging wounds), the total (n) varies due to missing values.

Variables		Total* (n=154)	Filarial IgG positivity		p-Value
			Yes, n=16 (10.4%)	No, n=138 (89.6%)	
Gender	Male	44 (28.6%)	4 (25.0%)	40 (29.0%)	0.74
	Female	110 (71.4%)	12 (75.0%)	98 (71.0%)	
Age (years), mean±SD		54.70±15.6	55.13±15.21	54.66±15.69	0.91
Age groups	≤10	1 (0.6%)	0 (0.0%)	1 (0.7%)	1.00
	11-24	6 (3.9%)	0 (0.0%)	6 (4.3%)	
	25-49	45 (29.2%)	5 (31.3%)	40 (29.0)	
	≥50	102 (66.2%)	11 (68.8%)	91 (65.9%)	
Education	None	79 (51.3%)	10 (62.5%)	69 (50.0%)	0.49
	Primary	68 (44.2%)	6 (37.5%)	62 (44.9%)	
	Secondary	7 (4.5%)	0 (0.0%)	7 (5.1%)	
Marital status	Single	44 (29.1%)	4 (25.0%)	40 (29.6%)	0.75
	Married	71 (47.0%)	7 (43.8%)	64 (47.4%)	
	Separated/divorced	26 (17.2%)	3 (18.8%)	23 (17.0%)	
	Cohabiting	3 (2.0%)	1 (6.3%)	2 (1.5%)	
	Widowed	7 (4.6%)	1 (6.3%)	6 (4.4%)	
Tribe	Munyankole	3 (1.9%)	1 (6.3%)	2 (1.4%)	0.42
	Mukiga	132 (85.7%)	13 (81.3%)	119 (86.2%)	
	Others	19 (12.3%)	2 (12.5%)	17 (12.3%)	
Religion	Catholic	64 (42.1%)	8 (53.3%)	56 (40.9%)	0.57
	Protestant	66 (43.4%)	7 (46.7%)	59 (43.1%)	
	Moslem	1 (0.7%)	0 (0.0%)	1 (0.7%)	
	Pentecostal	11 (7.2%)	0 (0.0%)	11 (8.0%)	
	Others	10 (6.6%)	0 (0.0%)	10 (7.3%)	
Occupation	Housewife	9 (5.9%)	1 (6.7%)	8 (5.8%)	0.97
	Business	8 (5.3%)	1 (6.7%)	7 (5.1%)	
	Peasant farmer/grazing	114 (75.0%)	12 (80.0%)	102 (74.5%)	
	Student	2 (1.3%)	0 (0.0%)	2 (1.5%)	
	Civil servant	1 (0.7%)	0 (0.0%)	1 (0.7%)	
	Others	18 (11.8%)	1 (6.7%)	17 (12.4%)	
Residence type	Rural	137 (91.3%)	14 (93.3%)	123 (91.1%)	0.77
	Semi-urban/trading centre	13 (8.7%)	1 (6.7%)	12 (8.9%)	
Limbs affected	Lower limbs	149 (96.8%)	16 (100.0%)	133 (96.4%)	0.74
	Upper limbs	2 (1.3%)	0 (0.0%)	2 (1.4%)	
	All limbs	3 (1.9%)	0 (0.0%)	3 (2.2%)	
Discharging wounds	Yes	16 (11.3%)	0 (0.0%)	16 (12.7%)	0.14
	No	125 (88.7%)	15 (100.0%)	110 (87.3%)	

## Discussion

The study investigated cases of lymphedema in the Kamwenge district, Western Uganda, focusing on both filarial-related and non-filarial forms of the condition. Serological testing revealed that 10.4% (n=16) of participants were positive for filarial antibodies, all of whom had IgG, indicating past or chronic exposure. In contrast, only 1.9% (n=3) tested positive for IgM antibodies, suggesting a low rate of recent infections. The positivity rate in this study is consistent with findings from previous research, likely due to the use of similar laboratory methods and study designs [17]. However, it appears lower than rates reported in some other communities, which may reflect the impact of ongoing control measures such as Mass Drug Administration (MDA) with ivermectin and albendazole, as well as the widespread use of insecticide-treated mosquito nets. These interventions are known to reduce transmission in endemic areas [17,18].

Conversely, the majority of participants tested negative for filarial antibodies, suggesting the potential presence of non-filarial elephantiasis, specifically podoconiosis. This aligns with earlier studies conducted in Busiriba sub-county, the Kamwenge district, which confirmed the existence of podoconiosis in the region [4,19].

The identification of confirmed cases of filarial elephantiasis in the Kamwenge district indicates that the burden of elephantiasis in the region extends beyond podoconiosis and is further complicated by the presence of lymphatic filariasis [4]. Prolonged exposure of bare feet to irritant volcanic soils, especially without protective footwear, remains a significant risk factor for podoconiosis [19,20]. These findings underscore the need for further research into the genetic and chemical contributors to podoconiosis. To address both forms of elephantiasis effectively, key recommendations include promoting health education on the transmission of filarial worms, encouraging early diagnosis, ensuring proper foot hygiene, and advocating for the consistent use of protective footwear. Public awareness campaigns and community education on prevention and control strategies for both filarial and non-filarial elephantiasis are also essential in mitigating the health burden in the region.

Limitations

Rapid diagnostic tests are limited by their inability to quantify antibody levels, differentiate between filarial species, and their susceptibility to false-positive or false-negative results. Although the presence of IgG and IgM suggests past exposure or recent infection, it does not confirm active disease. Accurate determination of current infection and differentiation from previous exposure requires antigen-based tests, such as circulating filarial antigen (CFA) assays. The study’s cross-sectional design restricts the ability to determine causality between filarial infection and lymphedema, as it captures data at only one point in time without reflecting disease progression.

## Conclusions

Serological testing revealed that a small proportion of participants were positive for filarial antibodies, with all positive cases exhibiting IgG, indicative of past or chronic exposure. In contrast, only a few individuals tested positive for IgM, suggesting recent infection. These results point to a low level of ongoing transmission but a notable history of exposure to lymphatic filariasis within the Kamwenge district. This highlights the continued public health relevance of the disease and underscores the need for sustained surveillance, early case detection, and targeted public health strategies to effectively manage filarial-associated lymphedema. Additionally, the findings support the utility of antibody seroprevalence testing as a valuable surveillance tool, particularly in regions nearing the elimination of lymphatic filariasis. Nonetheless, integrating antibody-based surveillance into national control programs requires further investigation to define thresholds that distinguish between active transmission and elimination status. Longitudinal studies tracking antibody levels before and after multiple rounds of mass drug administration (MDA) are essential for understanding the relationship between antibody and antigen responses and for determining whether antibody testing can reliably indicate infection and transmission dynamics.
